# Effect of microorganisms on degradation of fluopyram and tebuconazole in laboratory and field studies

**DOI:** 10.1007/s11356-023-25669-3

**Published:** 2023-02-06

**Authors:** Magdalena Podbielska, Małgorzata Kus-Liśkiewicz, Bartosz Jagusztyn, Ewa Szpyrka

**Affiliations:** Department of Biotechnology, Institute of Biology and Biotechnology, University of Rzeszów, Pigonia 1, 35-310 Rzeszow, Poland

**Keywords:** Microorganisms, Degradation, Fluopyram, Tebuconazole, Consumer safe, Maximum residue level

## Abstract

Nowadays, chemical pesticides are the most widespread measure used to control crop pests and diseases. However, their negative side effects prompted the researchers to search for alternative options that were safer for the environment and people. Pesticide biodegradation by microorganisms seems to be the most reasonable alternative. The aim of the laboratory studies was to assess the influence of *Bacillus subtilis* and *Trichoderma harzianum*, used separately and combined together, on fluopyram and tebuconazole degradation. In field studies, the degradation of fluopyram and tebuconazole after the application of the biological preparation in apples was investigated. The results from the laboratory studies show that the greatest decomposition of fluopyram and tebuconazole was observed in tests with *T. harzianum* in a range of 74.3–81.5% and 44.5–49.2%, respectively. The effectiveness of fluopyram degradation by *B. subtilis* was 7.5%, while tebuconazole inhibited bacterial cell growth and no degradation was observed. The mixture of microorganisms affected the degradation of fluopyram in a range of 8.3–24.1% and tebuconazole in a range of 6.1–23.3%. The results from the field studies show that degradation increased from 3.1 to 30.8% for fluopyram and from 0.4 to 14.3% for tebuconazole when compared to control samples. The first-order kinetics models were used to simulate the residue dissipation in apples. For the determination of pesticide residues, the QuEChERS method for apple sample preparation was performed, followed by GC–MS/MS technique. Immediately after the treatments, the maximum residue level (MRL) values for tebuconazole were exceeded, and it was equal to 100.7% MRL for the Red Jonaprince variety and 132.3% MRL for the Gala variety. Thus, preharvest time is recommended to obtain apples in which the concentration of pesticides is below the MRL and which can be recognized as safe for humans.

**Supplementary Information:**

The online version contains supplementary material available at 10.1007/s11356-023-25669-3.

## Introduction

Pesticides are employed as the predominant method used in agriculture practices to prevent and control both diseases and pests (Anket et al. 2019). The application of pesticides improves the quality of agricultural production and reduces crop losses (Tudi et al. 2021). However, the widespread use of pesticides poses a risk due to the presence of their residues in both agricultural products and the environment (Rani et al. 2021). In Poland, pesticides are mostly used in horticulture and vegetable farming, mainly to protect fruit and vegetables against fungal diseases (Szpyrka 2015; Szpyrka et al. 2016). To minimize losses caused by diseases, it is necessary to perform fungicide treatments. Innovative generations of pesticides are being introduced to resolve the contradiction between stable production yielding high-quality agricultural products and environmental pollution (Huang et al. 2018). SDHI (succinate dehydrogenase inhibitors), DMI (demethylation inhibitors), and QoI (quinone outside inhibitors) are the most important groups of fungicides for agricultural disease control (Sierotzki and Scalliet 2013). Fluopyram, belonging to the SDHI group of fungicides (IUPAC name *N*-[2-[3-chloro-5-(trifluoromethyl)-2-pyridyl]ethyl]-α,α,α-trifluoro-ortho-toluamide), is an active substance of fungicides with a broad spectrum of activity. It is biologically active at all stages of fungal growth, from spore germination to spore production. Its spectrum of activity covers several pathogens important for agriculture, including those that cause gray mold and powdery mildew affecting vegetables, pome, and stone plants (Villaverde et al. 2018). DMI fungicides, also called sterol biosynthesis inhibitors (SBI), are used against a broad spectrum of fungal pathogens. The fungal sterol (ergosterol) plays a very important structural and signaling role in fungal cell membranes. DMI fungicides inhibit sterol 14α-demethylation by selectively binding to cytochrome P450 sterol 14α-demethylase (CYP51). This combination prevents the enzyme from obtaining substrate and further activation of oxygen that participates in the methyl group (C-14) removal from the sterol molecule. This results in the depletion of ergosterol, the main component of fungal membranes, and a high accumulation of potentially toxic intermediate sterol compounds, thus modifying the structure and the function of the cell membrane (Claassen et al. 2022; Strickland et al. 2022). This group of fungicides includes tebuconazole (IUPAC name 1-(4-chlorophenyl)-4,4-dimethyl-3-(1H-1,2,4-triazol-1-ylmethyl)pentan-3-ol) with a broad spectrum of activity. It is highly effective against fungal pathogens of fruit and vegetable plants. It is used in the protection of apple trees against powdery mildew. In Poland, both active substances are available in the Luna Experience 400 SC preparation, recommended for protection against fungal diseases in orchards.

Pesticides can be degraded in various ways, which include physical, chemical, and physicochemical degradation. Pesticide decomposition also depends on the physicochemical properties of the active substance, the plant on which the preparation is applied, and the frequency of pesticide usage (initial concentration). However, the above action may result in the formation of secondary pollutants in the form of metabolites. Very often these metabolites are more toxic than the active substance itself (Nandhini et al. 2021). Therefore, the introduction of biopesticides to the market was an alternative to widely used pesticides, including products containing microorganisms (Książek-Trela and Szpyrka 2022).

The application of individual strains or a consortium of microorganisms is suggested to be an agricultural method that is more environmentally friendly; they are also considered the principal agents of xenobiotic degradation (Arbeli and Fuentes 2007). In integrated pest management, using pesticides in combination with biological products is considered to be a good practice in agricultural production (Piwowar 2021). These strategies lead to higher nutrient content, reduce the negative impact on the environment, and potentially enhance consumer health benefits (Niu et al. 2021). Furthermore, they reduce the number of pesticide applications, broaden the spectrum of pest control, enhance plant nutrition, and mitigate soil pollution. Microorganisms can support the native flora of soils and plants in faster degradation of pesticides in the environment. In nature, there is a large number of microorganisms with a high level of adaptability and different types of metabolism that can use synthetic organic substances as a source of carbon, nitrogen, and energy for their growth (Gartner et al. 2012). At the same time, they can completely mineralize or degrade the organic pesticide into small, nontoxic molecules through various metabolic pathways (Levio-Raiman et al. 2021) (Li et al. 2017). Microbes, bacteria, actinomycetes, and fungi are used to effectively remove or detoxify pesticides, PCBs, and polycyclic aromatic hydrocarbons. The main types of microorganisms with high biodegradability potential include *Bacillus*, *Pseudomonas*, *Flavobacterium*, *Micrococcus*, *Acinetobacter*, *Aerobacter*, *Alkaligens*, *Burkholderia*, and *Sphingomonas* (Moscoso et al. 2013; Satish et al. 2017). Fungi with the potential for xenobiotics degradation include *Trichoderma*, *Fusarium*, *Aspergillus niger*, *Penicillium*, and *Oxysporum* (Marco-Urrea et al. 2015)*.*

The aim of the study was (i) to determine the efficiency of degradation of fluopyram and tebuconazole in the laboratory conditions after exposure to *Bacillus subtilis* (bacteria) and *Trichoderma harzianum* (fungi) and a mixture of bacterial and fungal cultures; (ii) to verify in the field conditions if the application of biological preparations containing microorganisms affected the content of fluopyram and tebuconazole residues in apples. An additional aim was to determine the dissipation kinetics of fluopyram and tebuconazole residues in apples and the safety of this fruit for the consumer.

## Material and methods

### Experiments under laboratory conditions

The biological preparation used in field tests contained strains of *Bacillus subtilis* bacteria and the fungi *Trichoderma harzianum* and Glomus spp. As the manufacturer does not provide the detailed composition of the commercial preparation, reference strains of *Bacillus subtilis* PCM 486 and *Trichoderma harzianum* KKP 534 were selected for laboratory tests.

### Strain, media, and culture conditions

*Bacillus subtilis* PCM 486 and *Saccharomyces cerevisiae* SP4 (obtained from the Culture Collection of the University of Rzeszow, Poland), and *Trichoderma harzianum* KKP 534 (obtained from the Institute of Agricultural and Food Industry, Poland), were inoculated into tubes containing specific medium. Bacteria cells were incubated in a nutrient broth (NB, BTL, Poland), composed of meat extract (2 g/L), yeast extract (2 g/L), peptone (5 g/L), NaCl (4 g/L), and glucose (10 g/L). Glucose potato broth (PDB; potato extract = 4 g/L and glucose = 20 g/L) was used for preculturing of *Saccharomyces cerevisiae* and *Trichoderma harzianum*. The cultures were incubated for 18 h at 37 °C for bacteria and 30 °C for fungi with shaking at 150 rpm (Incubator MaxQ 6000, Thermo Fisher Scientific, Waltham, MA, USA). Tested cultures were inoculated at an initial OD_600_ of 0.05–0.1 from precultures in the mid-exponential growth phase and were performed for further experiments. All media were purchased from BTL (Warsaw, Poland) and Sigma-Aldrich (Burlington, MA, USA). Phosphate buffered saline (1 × PBS; 137 mM NaCl, 2.7 mM KCl, 10 mM Na_2_HPO_4_, and 1.8 mM KH2PO4) was used for serial dilutions.

### Determination of the concentration inhibitory effect for *B. subtilis* and a reference yeast, *S. cerevisiae*

The minimum inhibitory concentration (MIC) test was used to verify the inhibitory effect of fluopyram and tebuconazole concentrations on *B. subtilis* and *S. cerevisiae* strains. The MIC assay was done according to the standard protocols (CLSI 2010). The inoculum was prepared as described above (“Experiments under laboratory conditions” section). Briefly, bacteria and yeast precultures were diluted to match the optical density of OD 600 ~ 0.1. A total of 100 µL of cell suspensions were applied onto the 96-well microtiter plate. The fluopyram (Supelco Inc., Bellefonte, PA, USA) and tebuconazole (Instytut Przemysłu Organicznego, Warsaw, Poland), dissolved in methanol, were added to selected wells at concentrations ranging from 0.02 to 1000 µg/mL. The microtiter plates were incubated for 24 h at 37 and 30 °C for bacteria and yeast strains, respectively, in a shacking incubator (Biosan, Riga, Latvia). All the measurements were conducted in triplicate. The negative control was the pure culture medium, and the positive control were strains of *B. subtilis* and *S. cerevisiae* not treated with fluopyram and tebuconazole. The absorbance was measured at “zero” time and after 24 h, at a wavelength of 600 nm (Infinite M200, Tecan Group Ltd., Männedorf, Switzerland). The lowest concentration of the tested suspensions, which was transparent, was considered the MIC. The minimum bactericidal concentration (MBC) and minimum fungicidal concentration (MFC) tests were performed for substances showing growth-inhibitory effects on bacteria and yeast, respectively.

#### Evaluation of minimum bactericidal/fungicidal concentration

For this purpose, cultures from a relevant well of the plate, in which the inhibition of bacterial and fungal growth was observed, were seeded on plates with solid medium (glucose potato broth solidified with agar (20 g/L)). Plates were incubated for further 24 h at 37 and 30 °C for bacteria and yeast strains, respectively. The complete lack of growth of cells exposed to the relevant concentration was recorded as a lethal value for the tested microorganism.

#### Fluopyram and tebuconazole degradation by *B. subtilis*, *T. harzianum*, and consortium of the strains

The pesticide degradation assay was performed in a 100-mL flask containing tenfold diluted NB, PDB medium, or both, supplemented with fluopyram and tebuconazole at a final concentration of 100 µg/mL. The flasks were incubated on a rotatory shaker at 100 rpm at 30 ± 2 °C for bacteria and 28 ± 2 °C for fungi and the mixture of microorganisms. Non-inoculated solutions of fluopyram and tebuconazole diluted in NB and PDB media were used as control samples. All tests were carried out in triplicate. To determine fluopyram and tebuconazole concentrations during the incubation, aliquots were periodically collected from the flasks under sterile conditions on days 0, 3, 5, 7, and 14 of the experiment. The samples were collected in polypropylene tubes and analyzed immediately. Additional samples were collected from the flasks in which fluopyram and tebuconazole were degraded by *B. subtilis* for the evaluation of the cellular metabolic activity.

#### Evaluation of metabolic activity of bacteria cells

To assess the metabolic activity of bacteria cells, the bacteria viability was checked using the redox indicator AlamarBlue (Hieke and Pillai 2018). Briefly, 180 µL of the culture was applied onto the black 96-well plate. Next, 20 µL of AlamarBlue reagent (0.01%) (Thermo Fisher Scientific, Waltham, MA, USA) was added, and then the plate was incubated in the dark for 10 min. The fluorescence at an excitation wavelength of 560 nm and an emission of 590 nm was measured with the Tecan INFINITE 200 Pro microplate reader (Tecan Group Ltd., Männedorf, Switzerland). The metabolic activity was expressed as a relative fluorescence unit (RFU).

### Experiments under field conditions

The supervised trials were carried out in two orchards Rzeszów and in Józefów on the Vistula. The field tests were carried out in 2017 and 2018 in two apple varieties, Gala and Red Jonaprince. In both orchards, standard agricultural activities were carried out throughout the growing season, including tree pruning, fertilization, and care treatments, in line with the principles of good agricultural practice.

#### Application of chemical and biological preparations

Treatments with a chemical preparation—Luna Experience 400 SC—at recommended label doses of 0.75 L/ha were performed in the entire plot with the variety (approx. 0.5 ha), 3 weeks before harvesting ripe apples. Luna Experience 400 SC contains two active substances: fluopyram 200 g/L (a compound from the anilide group) and tebuconazole 200 g/L (a compound from the triazole group). It is a fungicide for professional use in the form of a concentrated suspension to be diluted with water. The product has a systemic action for preventive or interventional use in the protection of apple trees against apple scab, apple mildew, and storage diseases: bitter rot, storage scab, gray mold, brown rot, and blue mold (wet rot). The apple trees of Red Jonaprince and Gala varieties were sprayed on August 23, 2017, and August 28, 2018, respectively. In each orchard, Luna Experience 400 SC was applied individually using a sprayer, Turbine N TNC 1000 (Annovi Reverberi, Modena, Italy), and Agrola 1500 (Zakład Handlowo-Produkcyjny AGROLA, Płatkownica, Poland) for Red Jonaprince and Gala, respectively. A biological preparation used in our studies was Zumba Plant® (NaturalCrop, Warsaw, Poland). The Zumba Plant is an organic fertilizer containing strains of microorganisms (*Bacillus* spp., *Trichoderma* spp.) and fungi (*Glomus* spp.). The three strains of beneficial microorganisms contained in the fertilizer interact with each other. The product stimulates plant growth, especially of the roots as well as the above-ground parts of plants. Zumba promotes fertilization of the soil (Zumba Plant label). Apple trees of Red Jonaprince and Gala varieties were sprayed on August 30, 2017, and September 4, 2018, respectively.

Degradation studies had a form of comparative experiments. The chemical preparations were applied to the whole experimental plot (about 0.5 ha) of a given variety. On the seventh day of the experiment, the plot was divided into two parts, each containing 6 rows. Part I was the control, where plants were sprayed with water. In part II, plants were sprayed with biological preparation.

#### Weather conditions

**Fig. 1:**
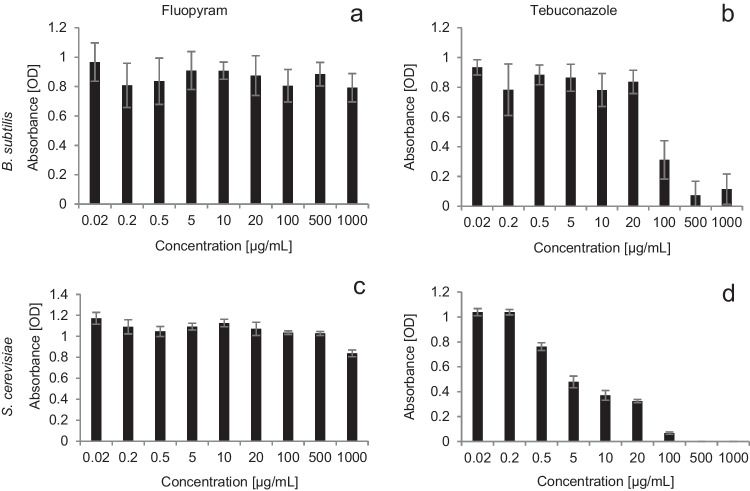
Inhibition of *B. subtilis* growth after 24 h exposure to fluopyram (**a**) and tebuconazole (**c**), and inhibition of *S. cerevisiae* growth after 24 h exposure to fluopyram (**b**) and tebuconazole (**d**). Data were expressed as the mean ± standard deviation, *n* = 3. The wavelength was 600 nm

In Józefów, the weather condition was measured using the WatchDog 2900ET weather station (Spectrum Technologies, Inc., Aurora, IL, USA) installed in the orchard. For the Rzeszów region, temperature and rainfalls were taken from the site https://www.weatheronline.pl/. Temperature and rainfall were recorded for 3 weeks during each experiment (Supplementary Information (SI) Fig. 1a, b).

#### Collection of apple samples

The representative apple samples from each variety were collected at 12 h and on days 5, 8, 12, 14, and 21 after the Luna Experience treatment. On each first two sampling dates, 4 samples consisting of 10 apples were collected from the entire experimental plot. On day 7, biological preparation was applied in part II of the experimental plot, and samples were collected for 12 h, and then on days 12, 14, and 21 after the treatment. Each time, 4 control samples were collected from part I, and 4 test samples were picked from part II. According to Regulation 2007, samples of fruit were collected manually from randomly selected rows of trees, one apple from one tree in 4 replications (The Ministry of Health 2007).

### GC–MS/MS analysis

#### Standards and chemicals

The acetonitrile, acetone, and sodium chloride were provided by Honeywell (Charlotte, NC, USA). Petroleum ether, magnesium sulfate anhydrous, sodium sulfate anhydrous, trisodium citrate dihydrate (Na_3_C_6_H_5_O_7_·2H_2_O), were from Chempur (Piekary Śląskie, Poland), and disodium hydrogen citrate sesquihydrate (Na_2_HC_6_H_5_O_7_·1.5H_2_O) was purchased from Sigma-Aldrich (Burlington, MA, USA). A primary secondary amine (PSA, a polymerically bonded ethylenediamine-N-propyl phase that contains both primary and secondary amines, 50 µm particle size, 70 Å pore size), internal standard triphenyl phosphate (TPP, molecular formula: C_18_H_15_O_4_P, molecular weight: 326.3 g/mol, CAS No: 115–86-6), and fluopyram were from Supelco (Bellefonte, PE, USA), and tebuconazole was supplied by Instytut Przemysłu Organicznego (Warsaw, Poland). The purity of all standards was above 98%. Fungicide standards at the concentration of 1000 µg/mL were prepared in acetone and stored at − 17 °C. Working solutions were prepared by dissolving appropriate amounts of the standard solution in petroleum ether and stored at 4 °C.

#### Laboratory samples preparation procedure

A total of 0.1 mL of culture medium samples were added to a 15-mL centrifuge tube, followed by 5 mL of acetone and 0.5 g of anhydrous sodium sulfate. Then, the tubes were shaken vigorously for 1 min (BenchMixerTM, Benchmark Scientific, Inc., Edison, NJ, USA) and centrifuged at 3500 rpm for 5 min (MPW-350R, MPW MED. INSTRUMENTS, Warsaw, Poland). A total of 0.2 mL of upper phase were added to a screw-cup vial containing 0.8 mL of petroleum ether and 0.1 mL of internal standard. The extracts were then analyzed using GC–MS/MS.

#### Apple samples preparation procedure

The sample preparation method was based on pesticide multiresidue determination method standards (the Polish Committee for Standardization 2018). The laboratory apple samples were homogenized to generate a uniform sample that is representative of the product. A total of 10 g of homogenate were weighed into a 50 mL centrifuge tube. In the extraction process, 10 mL of acetonitrile were added to samples, and then the tube was shaken vigorously for 1 min (BenchMixerTM, Benchmark Scientific, Inc., Edison, NJ, USA). Then, buffer salts containing 4 g of magnesium sulfate, 1 g of sodium chloride, 1 g of trisodium citrate dihydrate, and 0.5 g of disodium hydrogen citrate sesquihydrate were added. Then samples were shaken vigorously for 1 min and centrifuged at 3500 rpm for 5 min (MPW-350R, MPW MED. INSTRUMENTS, Warsaw, Poland). In the clean-up process, 5 mL of the organic layer was transferred to a 15 mL polypropylene tube containing 0.9 g of magnesium sulfate and 0.15 g of PSA. The tubes were shaken for 30 s and centrifuged at 3500 rpm for 5 min. A total of 0.75 mL of the clean extract were transferred into a screw cup vial and evaporated under a stream of nitrogen. The dry extract was dissolved in 0.75 mL of petroleum ether, and 0.1 mL of the internal standard was added. The extracts were analyzed using GC–MS/MS.

#### Instrumental analysis GC–MS/MS

The quantification of penthiopyrad was performed using the 7890A gas chromatograph (Agilent Technologies, Palo Alto, CA, USA) equipped with a mass detector, model 7000 (GC–MS/MS QQQ). The injector was in the splitless mode at a temperature of 250 °C, with an injection volume of 1.0 μL, and using helium (99.99999 purity, flow 1 mL/min) as the carrier gas. Chromatographic separations were conducted using a HP-5 Ultra Inert MS column (30 m × 0.25 mm I.D. × 0.25-μm). The column temperature was maintained at 40 °C for 2 min, then increased to 220 °C at 30 °C/min, increased to 260 °C at 5 °C/min, and increased to 280 °C at 20 °C/min, and maintained for 8 min. The MS/MS system was equipped with an electrospray ionization source (ESI). The electron ionization mode of − 70 eV, with temperatures of 250 °C for the transfer line, 230 °C for the ion source, and 150 °C for quadrupoles, was applied. Fluopyram and tebuconazole were detected in the multiple reaction monitoring modes (MRM). In the qualitative analysis, monitored ion transitions were 173.0 → 145.1 (5), 250.1 → 125.0 (20), and 326.1 → 128.0 (15) for fluopyram, tebuconazole, and the internal standard, respectively. In the quantitative analysis, they were 396.1 → 223.0 (10) for fluopyram, 250.1 → 70.1 (10) for tebuconazole, and 326.1 → 228.4 (15) for TPP. Mass Hunter Software (version B.07.06, Agilent Technologies, Palo Alto, CA, USA, 2010) was used for instrument control and data acquisition and processing.

#### Method validation

The methods were validated in accordance with the requirements of the European Commission contained in the document SANTE/12682/2019 (European Commission DG Health and Food Safety 2019). Linearity, accuracy (expressed as recovery), precision (expressed as RSD), and LOQ were determined. The validation study was conducted using apples and broth samples previously checked to be free of fluopyram and tebuconazole residues. Matrix-matched calibration standards at concentrations of 0.01, 0.05, 0.1, 0.5, and 1 µg/mL were prepared to obtain calibration curves. Linearity was determined on a basis of coefficients of determination (*R*^2^). Recovery studies were based on a calculation of recoveries in two concentration levels and five repetitions in the matrix. The accuracy and precision were assessed and expressed as an average recovery and a relative standard deviation (RSD), respectively. The limit of quantification (LOQ) was defined as the minimum quantified concentration. The limit of detection (LOD) was determined according to the signal-to-noise ratio criteria (S/N); LOD = 3 S/N.

### Dissipation kinetic of fluopyram and tebuconazole in the field trial

The fluopyram and tebuconazole dissipation in apples was evaluated according to the first-order kinetics regression. The dissipation trends in apples were calculated according to the following equation: *P*_t_ = *P*_0_ × *e*^−*kt*^, where *P*_t_ represents the residue concentration at the time *t* (mg/kg), *P*_0_ represents the initial residue concentration at the time zero, *t* = 0 (mg/kg), *t*—time, and *k* represents the degradation rate constant, as days. From the above formula, the half-life for tested active ingredients was calculated using the formula: *t*_1/2_ = ln2 *k*^−1^.

### Statistical analysis

Values of fluopyram and tebuconazole residues in the dissipation kinetics and the degradation determinations are expressed as means. Mean values were calculated from three independent experiments in laboratory studies and four experiments in field studies. The statistical significance was assessed using Microsoft Office Excel, using the Student’s *t*-test for independent samples with the two-trace distribution. The statistically significant differences were noticed in the charts as * *p* < 0.05, ** *p* < 0.01, and ***p < 0.001.

## Results

### Methods validation

The linearity of the method at the concentration range of 0.01–1 mg/kg was obtained. All standard curves exhibited a good linear relationship with a correlation coefficient *R*^2^ ≥ 0.99.

The spiked recoveries at different concentration levels in different matrices were used to represent accuracy. The recoveries were 74.2–92.1%, 90.4–91.5%, and 106.0–114.4% for fluopyram, and 86.1–110.8%, 83.7–105.2%, and 90.8–109.4% for tebuconazole, in NB, PBD and apple samples, respectively. The RSDs of spiked sample recoveries were used to describe precision. The corresponding standard deviation were 3.6–4.0%, 8.7–13.9%, and 1.9–3.3% for fluopyram, and 6.6–9.9%, 3.0–4.6%, and 2.2–3.5% for tebuconazole in NB, PBD, and apple samples, respectively. Intra-day and inter-day precisions expressed as RSD were below 10%. LOQ at the level of 0.01 mg/kg was quantified with acceptable accuracy and precision. The obtained validation results meet the requirements specified in the SANTE document (mean recoveries in the range of 70–120% and RSD ≤ 20%).

### Experiments under laboratory conditions

#### Inhibition growth and a lethal effect on *B. subtilis* and the reference fungus *S. cerevisiae* in response to fluopyram and tebuconazole

The minimum inhibitory concentrations of the fluopyram and tebuconazole for *B. subtilis* and *S. cerevisiae* were assessed by culturing cells on NB or PDB medium containing the relevant chemical at a concentration ranging from 0.02 to 1000 µg/mL. After 24 h of *B. subtilis* and *S. cerevisiae* exposure to the tested concentration range, no negative effect of fluopyram on the microorganisms’ growth rate was observed (Fig. 1a, b). It can be concluded that this chemical does not inhibit the growth of the strains; no MIC or MBC values were determined. On the contrary, tebuconazole inhibited bacterial and yeast cell growth, which was reflected in the decreased absorbance value in Fig. 1. On a basis of these results, *B. subtilis* had a MIC value of 100 µg/mL (Fig. 1c), while *S. cerevisiae* at concentrations amounted to 0.5 µg/mL (Fig. 1d).

**Fig. 2:**
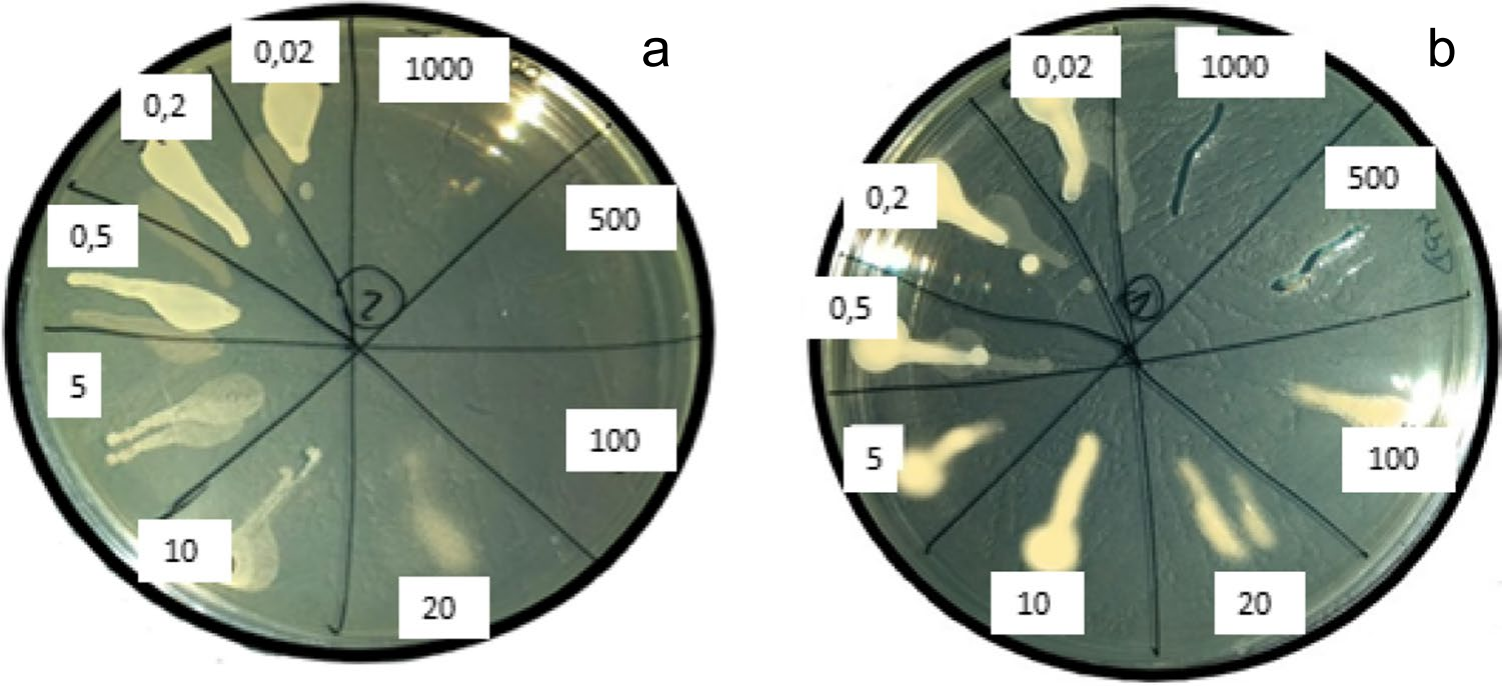
Exposure of *B. subtilis* (**a**) and *S. cerevisiae* (**b**) to tebuconazole (0.02–1000 µg/mL) for 24 h; MBC test (**a**), MFC test (**b**)

It must be noted that the decrease in absorbance does not clearly indicate a lethal effect. Therefore, samples showing inhibition of growth by tebuconazole were further cultured by seeding them onto plates with the appropriate medium and incubating them for further 24 h. After this time, the growth intensity was assessed. In the case of tebuconazole, along with an increase in its concentration in the culture, the growth intensity decreased until it was completely absent. Tebuconazole concentrations at a level lethal for the bacteria or the fungi are understood as no growth on the agar plate. *B. subtilis* showed MBC values of 100 µg/mL (Fig. 2a). The MFC value for S. cerevisiae is at a concentration of 500 µg/mL (Fig. 2b).

#### Decomposition of fluopyram and tebuconazole exposed to microbial strains, fungi, and mixture culture of *B. subtilis* and *T. harzianum*

**Fig. 3:**
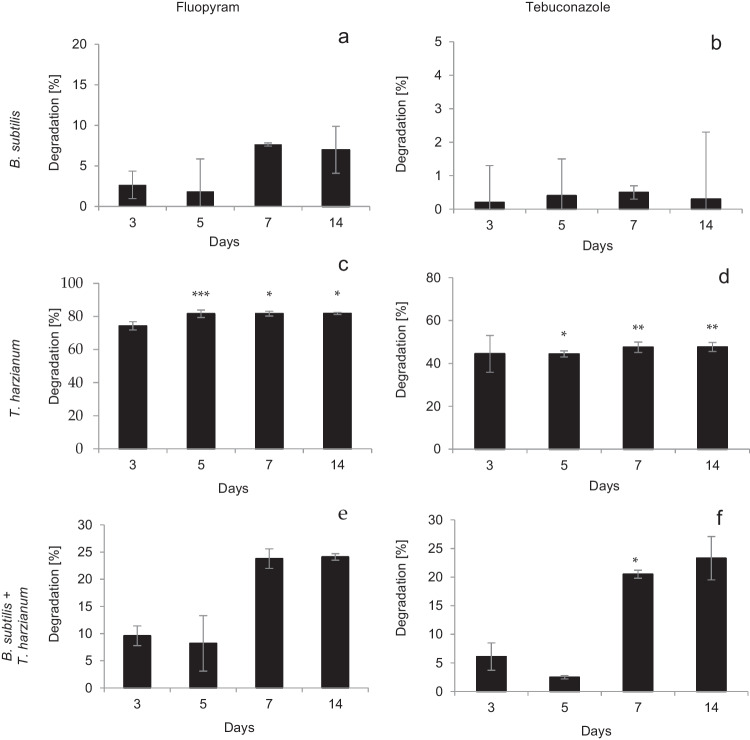
Degradation of fluopyram (**a**) and tebuconazole (**b**) treated with *B. subtilis*. Degradation of fluopyram (**c**) and tebuconazole (**d**) treated with *T. harzianum*. Degradation of fluopyram (**e**) and tebuconazole (**f**) was treated with a mixed culture of *B. subtilis* and *T. harzianum*. Data were expressed as the mean ± standard deviation, *n* = 3. The statistically significant differences were observed in the charts for * *p* < 0.05, ** *p* < 0.01, and ***p < 0.001

The results of the laboratory experiments concerning the microorganisms’ influence on the degradation of fluopyram and tebuconazole were assessed. Samples were taken under sterile conditions on days 0, 3, 5, 7, and 14 of the experiment. The highest degradation rate was observed in the tests where samples were subjected to degradation by fungal strains, i.e., *T. harzianum*. Fluopyram degradation was 74.3% on day 3 and approximately 81.5% on subsequent days (Table 1, Fig. 3c). For tebuconazole, the decomposition rate was in a range of 44.5–49.2% (Table 1, Fig. 3d). In the experiment with the application of the mixed culture of *B. subtilis* and *T. harzianum*, the fluopyram degradation ranged from 8.3 to 24.1% (Table 1, Fig. 3e). The percent degradation of tebuconazole was determined as ranging from 6.1% on day 5 to 23.3% on day 14 (Fig. 3f). The lowest degradation rate was observed in samples subjected to degradation by *B. subtilis* after application. The fluopyram residues decreased by 1.8–2.7% on days 3 and 5 and by 7.6% on days 7 and 14 when compared to the control samples (Table 1, Fig. 3a). The degradation of tebuconazole was in the range of 0.2–0.5% (Table 1, Fig. 3a).

**Table 1:** Concentrations of fluopyram and tebuconazole in samples treated with *B. subtilis*, *T. harzianum*, and mixed culture of microorganisms and in control samples

Day	Fluopyram concentration ± SD [µg/mL]			Tebuconazole concentration ± SD [µg/mL]		
	Control	After treatment	Loss %	Control	After treatment	Loss %
*B. subtilis*						
0	119.6 ± 3.8	120.5 ± 5.9	0	91.0 ± 3.6	90.5 ± 2.5	0
3	117.8 ± 1.8	114.6 ± 3.0	2.7	90.4 ± 2.3	90.2 ± 3.3	0.2
5	112.0 ± 0.7	109.9 ± 0.9	1.8	89.9 ± 2.3	89.5 ± 2.1	0.4
7	106.4 ± 3.7	98.3 ± 0.4	7.6	91.1 ± 1.1	90.6 ± 8.2	0.5
14	104.5 ± 11.0	97.2 ± 2.9	7.0	91.1 ± 5.0	90.8 ± 6.9	0.3
*T. harzianum*						
0	112.0 ± 2.1	112.5 ± 5.5	0	100.2 ± 3.4	100.9 ± 5.2	0
3	111.0 ± 4.2	28.5 ± 1.4	74.3	92.3 ± 12.2	51.1 ± 5.5	44.6
5	99.8 ± 15.0	18.5 ± 3.4	81.5	87.0 ± 4.8	48.3 ± 0.5	44.5
7	97.3 ± 13.9	17.8 ± 1.8	81.7	84.8 ± 3.0	44.5 ± 0.2	47.5
14	97.5 ± 14.8	17.8 ± 2.5	81.7	84.1 ± 4.1	42.8 ± 2.5	49.2
Mixed culture of *B. subtilis* + *T. harzianum*						
0	112.2 ± 6.0	112.0 ± 4.0	0.2	90.0 ± 4.3	90.4 ± 6.6	0
3	110.8 ± 4.8	100.0 ± 9.9	9.7	81.8 ± 7.6	76.8 ± 6.9	6.1
5	109.5 ± 10.8	100.4 ± 8.0	8.3	80.8 ± 3.9	74.3 ± 6.0	8.0
7	108.5 ± 7.8	82.8 ± 7.2	23.7	81.0 ± 1.2	64.5 ± 2.1	20.4
14	108.0 ± 12.6	82.0 ± 8.3	24.1	82.5 ± 10.4	63.3 ± 3.0	23.3

#### Estimation of *B. subtilis* cell viability

**Fig. 4:**
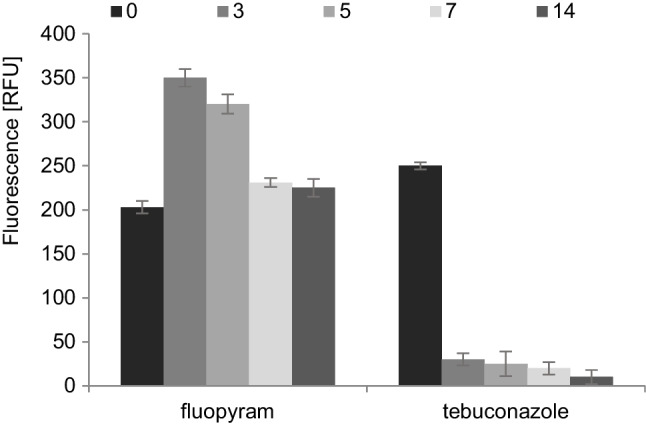
Bacterial cell viability in the presence of fluopyram and tebuconazole during 14 days of the experiment

The method based on luminescence measurements was used to determine the effect of fluopyram and tebuconazole on *B. subtilis* and *T. harzianum* viability. During the incubation of *B. subtilis* cells with fluopyram on day 3, an increase in the metabolic activity of the cells was observed, which indicated an increase in their number. With further cultivation on days 7 and 14, the metabolic activity decreased by 35%, which could be due to the reduced nutrient intake, the formation of harmful metabolites, and the inhibitory effect of the pesticides used. The complete inhibition of the cells’ metabolic activity had been observed for tebuconazole from day 3 of the experiment (Fig. 4).

### Experiments under field conditions

Chemical treatments in orchards in Józefów on the Vistula River and in Rzeszów were performed with the chemical preparation Luna Experience 400 SC at the recommended dose of 0.75 L/ha, containing the active substances fluopyram and tebuconazole in the amount of 200 g/L of each substance. The trial in apple varieties Red Jonaprince and Gala started on August 23, 2017, and August 28, 2018, respectively. Twelve hours after the treatments, the sampling started and was continued on August 28 and 31 and September 4, 7, and 13 for the Red Jonaprince variety, and September 2, 5, 9, 11, and 18 for the Gala variety. The biological preparation was applied in part II in both varieties 7 days after the application of the chemical preparation (more information about parts I and II, see “Application of Chemical and Biological Preparations”).

#### The fate of fluopyram and tebuconazole after application of the biological preparation Zumba Plant®

**Fig. 5:**
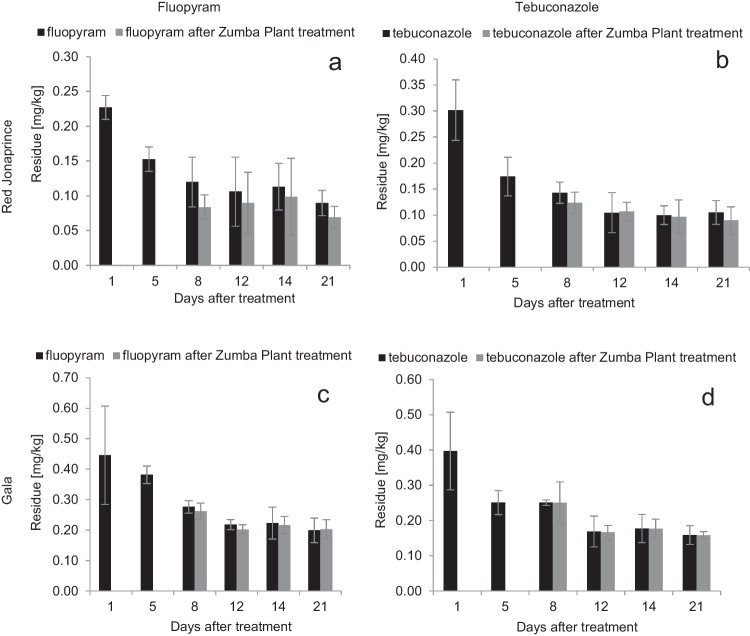
Fluopyram and tebuconazole residues after application of a chemical preparation Luna Experience 400 SC and a biological preparation Zumba Plant® in Red Jonaprince (**a**, **c**) and Gala (**b**, **d**) varieties, respectively. Data were expressed as the mean ± standard deviation, *n* = 4

After the application of the biological preparation in the Red Jonaprince variety, the fluopyram residue was at the level of 0.083 ± 0.018 mg/kg (Table 2, Fig. 5a). On the next two sampling dates, the residues decreased by ca 15% (Table 2). On the last sampling date, the residue of fluopyram amounted to 0.069 ± 0.016 mg/kg (Table 2, Fig. 5a). In the Gala variety, on the first sampling date, the fluopyram residues were at the level of 0.262 ± 0.027 mg/kg (Table 2, Fig. 5c), and on the second, third, and fourth sampling date were at the level of 0.202 ± 0.016 mg/kg, 0.223 ± 0.052 mg/kg, and 0.199 ± 0.040 mg/kg, respectively (Table 2, Fig. 5c).

**Table 2:** Sampling dates and fluopyram and tebuconazole concentrations after treatment with a chemical preparation Luna Experience 400 SC and a biological preparation Zumba Plant® in Red Joanprince and Gala varieties

Sampling date	Days after treatment with a chemical preparation	Fluopyram concentration ± SD [kg/mg]				Tebuconazole concentration ± SD [mg/kg]	
		Control	After treatment with a biological preparation	Loss %	Control	After treatment with a biological preparation	Loss %
Red Jonaprince							
8/24/2017	1	0.227 ± 0.017	–	0	0.302 ± 0.058	–	0
8/28/2017	5	0.153 ± 0.018	–	0	0.174 ± 0.037	–	0
8/31/2017	8	0.120 ± 0.036	0.083 ± 0.018	30.8	0.143 ± 0.020	0.124 ± 0.020	13.3
9/4/2017	12	0.106 ± 0.050	0.090 ± 0.044	15.1	0.105 ± 0.038	0.107 ± 0.018	− 1.9
9/7/2017	14	0.113 ± 0.034	0.098 ± 0.056	13.3	0.100 ± 0.018	0.097 ± 0.032	3.0
9/13/2017	21	0.090 ± 0.018	0.069 ± 0.016	23.3	0.105 ± 0.023	0.090 ± 0.026	14.3
Gala							
8/29/2018	1	0.446 ± 0.162	–	0	0.397 ± 0.110	–	0
9/2/2018	5	0.382 ± 0.029	–	0	0.251 ± 0.034	–	0
9/8/2018	8	0.276 ± 0.021	0.262 ± 0.027	5.1	0.251 ± 0.008	0.250 ± 0.059	0.4
9/9/2018	12	0.218 ± 0.017	0.202 ± 0.016	7.3	0.169 ± 0.044	0.167 ± 0.019	1.2
9/11/2018	14	0.223 ± 0.052	0.216 ± 0.028	3.1	0.177 ± 0.040	0.177 ± 0.027	0
9/18/2018	21	0.199 ± 0.040	0.202 ± 0.032	− 1.5	0.159 ± 0.027	0.158 ± 0.011	0.6

The residues of tebuconazole in the Red Jonaprince variety on the day following the application of the Zumba Plant® biological preparation amounted to 0.124 ± 0.020 mg/kg (Table 2). On the next two sampling dates, i.e., September 4 and 7, the residues in both the control samples and those with the applied microorganisms were at a similar level. On the last sampling date, the residues amounted to 0.090 ± 0.026 mg/kg. In the Gala variety, tebuconazole residues were approx. at the same level in relation to the control samples on all sampling dates.

#### Dissipation kinetics

**Fig. 6:**
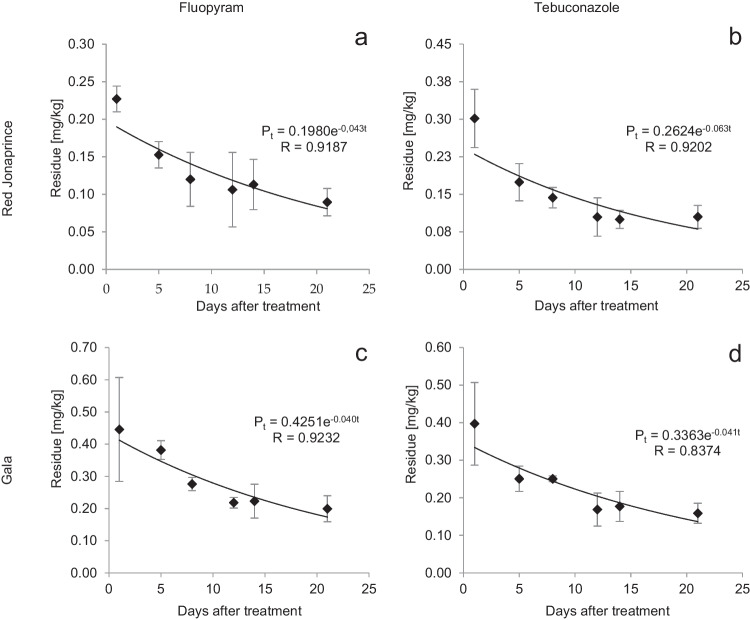
Dissipation kinetics of fluopyram (**a** and **c**) and tebuconazole (**b** and **d**) in Red Jonaprince and Gala varieties, respectively. Data was expressed as the mean ± standard deviation, *n* = 4

On the first day after Luna Experience 400 SC application, the fluopyram residues were at a level of 0.277 ± 0.017 mg/kg and 0.446 ± 0.162 mg/kg for Red Jonaprince and Gala, respectively (Table 2). The residues disappeared according to the exponential equations: *P*_t_ = 0.1980e^−0.043t^ (*R* = 0.9187) and *P*_t_ = 0.4251e^−0.040t^ (*R* = 0.9232) (Fig. 6a, c). At the time of the fruit harvesting, the residues were at the level of 0.090 ± 0.018 mg/kg in Red Jonaprince and 0.199 ± 0.040 mg/kg in the Gala variety (Table 2). The half-life was 16.1 and 17.3 days in Red Jonaprince and Gala, respectively.

The tebuconazole residues after the treatment were at the level of 0.302 ± 0.058 mg/kg, while on the day of the fruit harvest, they were at a level of 0.105 ± 0.023 mg/kg in the Red Jonaprince variety. In the Gala variety, they were at the level of 0.397 ± 0.110 mg/kg, and 21 days after the treatment, the residues were at the level of 0.159 ± 0.027 mg/kg (Table 2). The dissipation of the tebuconazole residue followed the equation: *P*_t_ = 0.2624e^−0.063t^ (*R* = 0.9202) in the Red Jonaprince variety, and *P*_t_ = 0.3363^e−0.041t^ (*R* = 0.8374) in the Gala variety (Fig. 6b, d). The calculated half-life was 11 and 16.9 days, respectively.

## Discussion

Fluopyram did not reduce the metabolic activity of *B. subtilis* cells. The slight inhibition of viability observed during the experiment resulted from the nutrient depletion, as well as from the gradual accumulation of metabolic products (Fig. 4). Tests evaluating the ability to culture microorganisms in a medium supplemented with fluopyram at concentrations ranging from 0.02 to 1000 µg/mL was performed. No inhibition of *B. subtilis* and *S. cerevisiae* growth was observed (Fig. 1a, b). In laboratory studies, a slight fluopyram degradation by *B. subtilis*, ranging from approx. 2% on days 2 and 5 to 7.5% on days 7 and 14 of the experiment, was observed (Fig. 3a). In the case of exposure to *T. harzianum*, the level of the active substance degradation ranged between 74.3 and 81.7% (Fig. 3c), while in the case of studies with mixed cultures of bacteria and fungi, the degree of degradation ranged from 8.3% on day 5 to 24.1% on the day 14 of the experiment (Fig. 3e), which may indicate a mutually inhibitory effect of these microorganisms (Kim et al. 2008). Naeem and Ahmad (2022) conducted a biodegradation test for fluopyram. They demonstrated the fluopyram degradation by three bacterial strains, *Streptococcus pneumoniae*, *Streptococcus pyogenes*, and *Escherichia coli*, at the level of 82.7%, 89.8%, and 99.3%, respectively. The fluopyram biodegradation by four fungi strains, *Aspergillus fumigatus*, *Aspergillus niger*, *Aspergillus flavus*, and *Aspergillus terreus*, was at the level of 24.2%, 90.7%, 91.3%, and 95.4%, respectively.

In studies of the *B. subtilis* bacteria cells, the viability in the presence of tebuconazole, a low cell viability was observed (Fig. 4). In the MIC test, the cell growth inhibition was observed after the *B. subtilis* exposure to tebuconazole at a concentration of 100 μg/mL (Fig. 1c). In the MBC test, it was shown that the tebuconazole concentration of 100 µg/mL has a lethal effect on bacterial cells (Fig. 2a). In the case of *S. cerevisiae*, the decrease in absorbance in the MIC assay was observed at a level as low as 0.5 µg/mL. In the MFC test, the lethality of the tested active substance was observed at concentrations of 500 and 1000 µg/mL (Fig. 2b)*.* These results are confirmed by other authors. Tebuconazole significantly changed population numbers and decreased microbial biomass and bacterial community diversity, and this decreasing trend became more pronounced with the increasing treatment frequency and concentration (Han et al. 2021; Baćmaga et al. 2021). The results of these tests are confirmed in our degradation studies. Tebuconazole was degraded by bacteria, but the degradation rate was only 0.2–0.5%, while when fungi were used, this rate reached the level of 44.5–49.2% (Fig. 3d). Considering the use of the mixed culture of bacteria and fungi with tebuconazole, a mutual inhibitory effect of bacteria and fungi was also observed. The degree of degradation ranged from 6.1 to 23.3% on day 14 of the experiment (Fig. 3e). The tebuconazole degradation degree of 16% was observed after the application of the yeast, *Candida parapsilosis*. The tebuconazole degradation was demonstrated to be at a level of 30% for *B. cereus* 2B9 and *Bacillus* sp. IB13, 35% for *Nocardia asteroides* LAB 911, and 50% for *Pseudomonas* sp. C12B ATCC, *Bacillus* sp. 29B3, and *Bacillus* sp. 3B6 (Youness et al. 2018). *Enterobacter sakazakii* and *Serratia sp.* showed the ability to degrade tebuconazole to 51% of its initial concentration (Sehnem et al. 2010). Within 21 days after *T. harzianum* application, tebuconazole was degraded by 68% (Obanda et al. 2008). Wang et al. (2018) reported that at a concentration of 500 mg/L, the degree of degradation was 64.11% and increased to 94.05% after using tebuconazole at a concentration of 200 mg/L. Four bacterial genera, *Methylobacterium*, *Burkholderia*, *Hyphomicrobium*, and *Dermacoccus*, were also identified as potential tebuconazole-degrading bacteria (Han et al. 2021). Tebuconazole is not degraded by *Geotrichum candidum* CBS 14,488, *Aureobasidium pullulans* G, and *Aspergillus niger* ATCC 9142 (Youness et al. 2018).

The field experiments were carried out with the use of the Luna Experience 400 SC preparation containing the active substances fluopyram and tebuconazole in the amount of 200 g of active substances/L. The research was carried out in two varieties of apple trees: Red Jonaprince (an orchard located in Józefów on the Vistula River) and Gala (an orchard located in Rzeszów) in a 2-year research cycle (2017 and 2018). The application of Luna Experience 400 SC was carried out at the recommended dose of 0.75 L/ha. Luna Experience 400 SC is a preparation with systemic action and therefore not very sensitive to washing. It is intended for preventive or interventional use against apple scab and apple mildew. In the case of storage diseases, it is used to protect fruit against bitter, brown, wet rot, storage scab, and gray mold.

Immediately after the application of the preparation, the fluopyram residues amounted to 0.227 ± 0.017 mg/kg in the orchard in Józefów on the Vistula River and 0.446 ± 0.162 mg/kg in the orchard in Rzeszów and decreased exponentially within 21 days to the levels of 0.090 ± 0.018 and 0.199 ± 0.040 mg/kg, respectively. The Maximum Residues Level (MRL) for fluopyram was set as 0.8 mg/kg (fluopyram MRL). Immediately after the treatment, the %MRL values of 28% for apples of the Red Jonaprince variety and 56% for apples of the Gala variety were obtained. After 21 days from application, the values represented 11% MRL and 25% MRL, respectively. This indicates that the apples are safe for consumption.

After treatments with the biological preparation, the degree of the fluopyram residue degradation differed slightly depending on the orchard. In Józefów, the initial fluopyram residues were degraded by 31% when compared to the control, then by 15%, and by 23% on the last sampling date (Fig. 5a). In Rzeszów, initially the degree of degradation of fluopyram was low and amounted to only 5% in relation to the control, and then on the next collection date it amounted to 7%. On the third sampling date, the fluopyram degradation rate was only 3%, and on the last date, no differences in residue levels were observed in the test and control samples (Fig. 5c). Undoubtedly, weather conditions are an important factor influencing the survival and activity of bacteria and fungi. In the orchard in Józefów on the Vistula, on the first sampling date, when the temperature was approx. 25 °C (favoring the growth of microorganisms) and no rainfall was recorded immediately after the application of the biological preparation, the degradation was faster than on the 2nd and 3rd sampling dates when the temperature was approx. 15 °C (SI Fig. 1a). In the orchard in Rzeszów, on the first sampling date, the degree of fluopyram degradation was only 5%, which could be caused by rainfall immediately after the treatment and partial washing off of the applied microorganisms (SI Fig. 1b). The low degree of degradation (7%) was observed later, and the lack of degradation on the subsequent dates confirming that their number of microorganism was so low (or they were completely absent) after washing off that there was no fluopyram degradation. In the literature, there are no data on fluopyram degradation by microorganisms in field crops.

The obtained results can only be compared with the previous studies on the disappearance of fluopyram in apples, as there are no studies on the degradation and the kinetics of the residues’ disappearance in the tested fruits under field conditions. For fluopyram, a half-life of 7.7 days was obtained (Podbielska et al. 2017). The obtained differences can be explained by the weather conditions during the experiments in 2017 and 2018 when air temperatures were about 5 °C lower than in 2016, which influenced the dissipation rate of the test substance.

Research by Patel et al. (2016) on the fluopyram behavior in onions showed that the half-life was 8.85 and 9.12 days for the standard dose (75 g/ha) and the double dose (150 g/ha), respectively. Lower initial deposits in the onion were reported by Sharma et al. (2022) and showed half-life values of 1.83 and 1.74 days after application at a level of 150 and 300 g/ha, respectively. In chili peppers, Saha et al. (2016) showed a half-life of 1.2 and 1.2 days for a single (100 g/ha) and a double dose (200 g/ha), respectively. In bell pepper fruit, the demonstrated half-life was 7.3 days (Yogendraiah Matadha et al. 2021). Dong and Hu (2014) showed that the half-life after fluopyram application in watermelon crops amounted to 6.48–6.60 days. Katna et al. (2018) reported a fluopyram half-life of 3.37 and 3.89 in French beans for the standard and double doses, respectively. The fluopyram dissipation study in mango was carried out after applications at the standard and double doses of 150 and 300 g/ha, respectively, and the determined half-life was 4.3–5.4 days (Mohapatra et al. 2018). The half-life of this active substance was 4.5–6.2 days in melon (Yizhi et al. 2020) and 36.48–57.76 days in banana soils (Zhou et al. 2021).

The tebuconazole residues after the application of the chemical preparation were at the level of 0.302 ± 0.058 mg/kg in the orchard in Józefów on the Vistula River and of 0.397 ± 0.110 mg/kg in the orchard in Rzeszów. MRL for tebuconazole was established to be at a level of 0.3 mg/kg (Tebuconazole MRL). Immediately after the treatments, the MRL values for tebuconazole were exceeded in both varieties, amounting to 100.7% MRL in Red Jonaprince and 132.3% MRL in Gala. A total of 21 days after the treatment, residues were at the level of 0.105 ± 0.023 mg/kg and 0.159 ± 0.027 mg/kg, which constituted 35% of MRL and 53% of MRL, respectively. After this time, apples were safe for consumption.

After the application of the biological preparation Zumba Plant® in the apple variety Red Jonaprince, the tebuconazole degree of degradation was 13%, while on two subsequent dates, the residues were at the same level in the test and control samples. On the last sampling date, the degree of degradation was 14% (Fig. 5b, d). The degradation of tebuconazole was similar to fluopyram, which confirms that weather conditions have a significant impact on the degradation of the active substances by microorganisms. In the apple variety Gala, no degradation of tebuconazole was observed after the application of the biological preparation. After the microorganisms were partially washed off, tebuconazole inhibited the microorganisms that remained on the plant, so no activity in the degradation of the tebuconazole residues was visible. Pan et al. (2022) indicate that the application of foliar fertilizer affected tebuconazole residues in cucumber. Wang et al. (2018) reported that after the application of the bacterium *Serratia marcescens* B1 on tebuconazole used in field and greenhouse trials in Chinese cabbage crops, the substance’s half-life was reduced by 15.93 and 23.09%, respectively. This data is consistent with the degradation results obtained in the Red Jonaprince variety.

In studies conducted in 2016, the determined half-life of tebuconazole was 7.9 days (Podbielska et al. 2017). A similar study was conducted by Patyal et al. (2013), who reported that the half-life values in apples ranged between 19.38–25.99 and 19.84–28.86 days for the application at a dose of 200 and 400 g of active ingredient/ha, respectively. Standard and double doses were used by Katna et al. (2018) in French beans, with a half-life value of 3.37 and 3.92 days, respectively. Patel et al. (2016) reported that the half-life of onion was 6.69–7.72 days, while Mohapatra et al. (2011) and Mohapatra (2014) showed the half-life of tebuconazole in this vegetable amounted to 1.7 days and 6 days, respectively. In a study by Saha et al. (2016), after the application of tebuconazole in chili peppers at a single (100 g/ha) and a double (200 g/ha) dose, the half-life was from 0.866 to 1.083 days, respectively. Also, Sahoo et al. (2012) obtained a half-life of 1 day in the same crop. In other crops, the value of the half-life was 6 and 3–3.8 days in mango (Mohapatra 2015; Mohapatra et al. 2018, respectively), 5.87–6.93 days in the watermelon (Dong and Hu 2014), 4.49 days in ginseng (Wang et al. 2015a), 0.9 days in tomato (Singh and Singh 2014), 2.8 days in strawberries (Wang et al. 2015b), 11.97 days in pepper (Fenoll et al. 2009), and 6.1 and 7.2 days in bell pepper leaves (Yogendraiah Matadha et al. 2021).

## Conclusions

In view of the obtained results described in this paper, it can be concluded that *B. subtilis* and *T. harzianum* may be potentially useful in the degradation of fluopyram and tebuconazole. The results obtained demonstrated that:

- *T. harzianum* efficiently degraded fluopyram and tebuconazole at the level of up to 81.5 and 49.2%, respectively.
- In the experiment with the application of the mixed culture of *B. subtilis* and *T. harzianum,* the degradation rate amounted to 24.1% for fluopyram and 23.3% for tebuconazole.
- The lowest degradation was observed for *B. subtilis* strains*.* Residue levels decreased by 7.6% for fluopyram and by 0.5% for tebuconazole.
- After the application of the biological preparation in the apple orchard, the residue levels decreased by 30% for fluopyram and by 14% for tebuconazole.
- Immediately after the application of pesticides, MRL for tebuconazole was exceeded (100.7% MRL in the Red Jonaprince variety and 132.3% MRL in the Gala variety).
- The preharvest interval of 21 days is necessary to obtain apples safe for consumption.

## Supplementary Information

Below is the link to the electronic supplementary material.

Supplementary file1 (DOCX 60 KB)

## Data Availability

No.

## Materials availability

No.
