# Supplementary material for: Effect of microorganisms on degradation of fluopyram and tebuconazole in laboratory and field studies
Source: Environ Sci Pollut Res Int. 2023 Feb 6;30(16):47727–41. doi: 10.1007/s11356-023-25669-3 (PMC10097794; doi:10.1007/s11356-023-25669-3)
Supplement: Supplementary file 1 — Supplementary file1 (DOCX 60 KB) [file 11356_2023_25669_MOESM1_ESM.docx]

a

**SI Fig 1.** Temperature and precipitation in a period from 8/23/2017 to 9/13/2017 during the experiment in the Red Jonaprince variety in Józefów nad Wisłą (a), and from 8/28/2018 to 9/18/2018 during the experiment in the Gala variety in Rzeszów (b)
